# Hospital and Institutional News

**Published:** 1918-06-29

**Authors:** 


### HOSPITAL AND INSTITUTIONAL NEWS.

### ATTEMPT TO COMMANDEER A HOSPITAL.

MR. F. J. FRANKAU, the Deputy Treasurer, presiding at a General and Special Court of Governors of St. George's Hospital, Hyde Park Corner, held last week, announced that a request had recently been made by the War Office for the entire surrender of the institution at a few days' notice for the treatment thereat of wounded officers. Further, the authorities in Whitehall reminded the governors of their powers under the Defence of the Realm Act to commandeer the building, but intimated that they preferred to give expression to their wishes in the form of a request. The house committee, in reply, pointed out that the institution was a charity established for the care of the sick-poor of London, and that it would be a breach of trust on their part to acquiesce in the demand of the Director-General. Nevertheless they expressed their willingness to place two hundred beds at the disposal of the War Office provided the remaining beds, 141 in number, were available for civilians. That offer, however, was not entertained and the request was subsequently withdrawn. It had been made, the Deputy Treasurer believed, because the War Office had been misled into the idea that the hospital wanted to move away, and that the building, in which the Director-General thought practically no work was being done for the sick-poor, was a "white elephant" to the house committee. That such was not so, he added, might be judged from the fact that last year 4,395 in-patients and 26,885 new out-patients had been cared for. The delusion under which the War Office apparently laboured showed a gross disregard to facts and to the poor.

### A HOSPITAL TREASURER'S PROPOSAL.

A GENEROUS decision of Mr. Alfred Corah, J.P., the treasurer of the Leicester Royal Infirmary, has been accepted with gratitude by the board. In consequence of the death of two of the trustees of the institution, it was found that numerous investments stood in the name of one trustee only, and all required to be dealt with in the transfer to new trustees. This applied likewise to the original conveyances and other documents of the institution, and would necessitate the expenditure of considerable time and money. For these and other reasons, which the treasurer thought would make for the more efficient administration of the infirmary, the securing of a charter of incorporation was to be recommended, and if the board approved, he (Mr. Corah) would, in order to carry out the proposal, personally bear the cost of the necessary application. Mr. Alfred Corah's work in augmenting the permanent income of the institution by securing a larger subscription list of several thousand pounds has on several occasions been referred to in THE HOSPITAL—his latest offer shows the great interest he continues to display in his duties as treasurer.

### RE-CHRISTENING A LONDON HOSPITAL.

THE Guardians of the City of London Lying-in Hospital have determined to change the name of the institution, and to re-christen it the City of London Maternity Hospital. It is alleged by the ladies' committee—we do not know on what ground —that the change is likely to increase the income. The English language is getting steadily more and more anæmic, and cumbrous modern euphemisms more and more seek to oust good old words and names which have the vice, to modern minds, of expressing their meaning. The words "lying-in" are to be found even in Tennyson; the excellent word "midwife" is threatened; an asylum is now a mental hospital; a curate an assistant priest; a privy a lavatory; and a lavatory a "toilet," whatever that may mean. It is a sorry business. These changes, being intended for the gross public, have a decidedly "moral" air. But, alas! in language and the arts progress is a crab which runs backwards.

### THE NEW CHAIRMAN OF CHELSEA HOSPITAL FOR WOMEN.

MR. T. DYER EDWARDES, J.P., has resigned the office of Chairman of the Council, which he has held for thirteen years. He has been a considerable benefactor to the hospital, and will remain a member of the Council. His successor is Sir F. W. R. Fryer, K.C.S.I., who for many years has been vice-chairman of the hospital.

### THE PROPOSED HOSPITAL AT THE CAPE.

IT is generally admitted that Cape Town is in want of new and increased hospital accommodation, and the matter presses because of the plan for a Medical School at Groote Schuur. A special committee of the Cape Hospitals Board having put the proposal to the Administrator to build a hospital with 200 beds on the Groote Schuur estate at a cost of £100,000, a deputation of all the authorities concerned, headed by the Administrator, has approached the Prime Minister to ask for the chosen site, a grant in aid of the scheme of £30,000, and a loan in advance of £20,000 to the Provincial Administration. The University of Cape Town will contribute the remaining £20,000. Unfortunately, the hospital question has been complicated by the expenditure in the recent past of large sums of money, which it is doubtful have achieved the purpose for which they were spent. In 1915 the Alexandra Hospital, Maitland, was completed at a cost of £160,000 for chronic and tuberculous patients. This hospital, which is said to have proved a white elephant, was quickly let to the authorities as a military hospital, but, in view of the shortage of hospitals in Cape Town, Sir Frederic de Waal suggested the further expenditure of £20,000 to convert the Alexandra into a general civil hospital. The medical profession opposed the suggestion, partly because it estimated the cost of conversion at four times the suggested sum, and partly because of its remoteness from the proposed Medical School at Groote Schuur. The latest scheme for a 200-bed hospital seems to show that the former plan of converting the Alexandra has been dropped. The decision is still in suspense, but, meantime, not only Cape Town, but Grahamstown and King Williamstown are in urgent need of hospital beds.

### A YEAR OF IMMORTALITY.

The second anniversary of the opening of the in-patient department of the South London Hospital for Women, Clapham Common, will be celebrated on July 20, when the Duchess of Sutherland will receive birthday gifts, including purses. Collectors of cash for the purses are encouraged to make strenuous efforts by an undertaking that a bed or cot will be named in honour of the subscriber who contributes, or the collector who obtains, £50, or £30 in annual subscriptions, respectively. Contributors or collectors of similar sums, not being annual subscriptions, can have a bed or a cot, as the case may be, named after them for one year. We doubt whether the privilege of naming a bed or a cot for a bare twelve months will much encourage collectors. The naming of beds can be overdone, and since its appeal lies precisely in the perpetuity of the donor's association with the hospital to which he subscribes, twelve months of "immortality" does not sound very attractive. Such a reward for assiduous collections sounds more suitable for the "white elephant" stall, which will be presided over by the Mayoress of Wandsworth on the occasion at the hospital. However, the course is commendable, and we hope that the success will be great.

### ZEEBRUGGE HEROES AND THE LONDON HOMŒOPATHIC.

The Earl of Donoughmore, a Vice-President and Treasurer of the London Homœopathic Hospital, occupied the chair at the annual meeting of the governors of that institution on the 12th inst. Since 1915 eighty-five beds have been placed at the disposal of the Admiralty for naval casualties which are being treated by the civilian medical and surgical staff of the hospital, in addition to the ordinary civilian patients. The maintenance of these eighty-five naval beds is a strain on the resources of the hospital, but is unavoidable. Sympathisers with sick and wounded sailors may be reminded that since the war began this hospital has held these beds at the disposal of the Admiralty, and already over 1,500 naval casualties from the battle of Jutland, from the Mediterranean, from Zeebrugge and elsewhere, have received surgical or medical treatment. The deficit on the year was £2,506. The board, therefore, is appealing for funds to liquidate a debt of £16,710. Zeebrugge should be a word to conjure with, and Mr. Attwood, the secretary, will no doubt make good use of his opportunity. By the way, Mr. W. Lee Mathews came up for election to the board last week. If we mistake not, he is an active member of the committee of the Stage Society. In such a capacity he might create a precedent by inducing the Society to give a performance on behalf of the appeal fund.

### PENSION DEDUCTION FOR SANATORIUM TREATMENT.

Letters in the daily press are often characterised by want of logic, a fact which recent correspondence in the *Glasgow Herald* shows well enough. "Tubercular" wrote complaining that disabled Service men at the sanatorium suffer a deduction of pension amounting to seven shillings a week. It should surely have been obvious that in these days the value of lodging, and of board on the extra rationing scale, for an adult male is at least five times as much as that sum, so that in the sanatorium, with seven shillings a week stopped, the tuberculous invalided soldier is much better off than outside it, where he has to keep himself. However, several people wrote round the subject, and this essential point remained without a trace of reference until "S" very sensibly remarked that the seven shillings were deducted for board, and that treatment was free. He noted further that a special increase of pension to 27s. 6d. was made as soon as the man was sent to the sanatorium, that the man's wife received 13s. 9d. per week, and children's allowances at the rate of 6s. 8d. for the first child, 5s. for the second, and 4s. 2d. for each succeeding child; so that a man with three children was kept free, and his family received a net income of £2 10s. 1d. per week. "Tubercular" had complained of the stoppage and mentioned instances of civilians who receive the same treatment free of cost. But he did not remember that it is only civilians with dependants who suffer no stoppages, that they have only, at most, 10s. a week to be stopped at all, and that this 10s. is surely earmarked for support of the family at home. If "Tubercular" does not complain of his pension outside the sanatorium, he should surely be satisfied with it when under institutional care.

### FARM COLONY FOR COMBATANTS.

The chairman and chief officers of the National Association for the Prevention of Consumption issue their annual appeal for funds for a Farm Colony for sailors and soldiers suffering from tuberculosis. Up to date the Association has received £21,000 for this purpose, and the sum required is £50,000, which, it is stated, will establish the colony. "Who so dead to patriotism, so lost in selfishness, who would deny to the war-shattered hero not only the necessities of his condition, but a debt of gratitude as well?" the appeal well says. It is a good object indeed—although probably the money might have been better spent in developing existing sanatoriums, in the direction of after-care and employment on the land, all over the country. But, like many superior things, that would not have sounded as well as the really inferior one. A St. Dunstan's for the tuberculous easily enters the comprehension, and therefore touches emotion. It is to be hoped it will also touch pockets.

### AN EAST-END HOSPITAL PROGRAMME.

THE East Ham Hospital is one of those with a future of inevitable expansion, and the parcel of land which has already been purchased at the back of the institution is likely to be added to. The neighbouring Durban House and grounds are now for sale, with the Town Council as one of the purchasers, and the president, Sir John Bethell, is now approaching the vendors to see if the hospital cannot acquire a portion of the site. Sir John hopes to raise part of the purchase-money in the City, and to leave the balance to be raised on the spot. The East Ham Hospital has at present twenty-five beds, while the population of the district approaches 200,000. A convalescent home, it appears, is needed, and Sir John Bethell does not seem to think that much difficulty will be found in raising the necessary money.

### THE INFLUENZA EPIDEMIC.

THE outbreak of influenza has become widespread, and has attacked soldiers and civilians, doctors and patients, private houses and hospitals with equal impartiality. The King George Hospital in Stamford Street has been one of the sufferers, and few parts of the country have escaped this minor but distressing disease. Even camp life does not seem to confer immunity. Why the present season, which has experienced no exceptional temperature or weather, should have been marked by this outbreak is not apparent, nor can the food rationing account for it. The rations are a nuisance, but no more; privation, even to meat-eaters, has not been felt.

### A TUBERCULOSIS POLICY FOR LONDON.

METROPOLITAN Borough Councils are endeavouring to remove some of the anomalies now in existence in the treatment of tuberculosis patients in London. At a recent conference, when twenty-one of the Metropolitan Borough Councils were represented, they passed a series of recommendations for the consideration of the Local Government Board. They suggested the formation of one central authority to be responsible for the control of all matters concerning tuberculous persons, whether insured or uninsured, and that the responsibility of recommending cases for treatment, whether in sanatoria or by other means, should be vested in and rest with the tuberculosis officer for each City or Borough Council, who was best able to judge of the treatment required through intimate knowledge of each patient's physical and home conditions. The conference was also of opinion that greater attention should be paid to the treatment of advanced and incurable cases. Most of these cases were in an infective state, but the only provision for them at present, if insured, was in a workhouse infirmary, which institution very few consented to enter. The segregation and housing of advanced cases should be undertaken by each City or Borough Council, who could provide its own suitable home. The conference further decided that the Metropolitan Boroughs, whether singly or in groups, should provide colony treatment, including employment and medical supervision, for suitable cases. Finally, the conference came to the conclusion that the time has arrived for day sanatoria or open-air schools to be provided for all districts, and that they should be regarded as preventative as well as curative institutions, and that attendance at them should not be confined to definite and notified cases of tuberculosis only. The recommendations, it will be seen, are drastic; but if carried out in their entirety they would help to reduce the disease.

### GRAVEL FOR THE WAR OFFICE.

THE Eton Rural District Council is protesting against the War Office proposal to remove its isolation hospital, which serves twenty parishes, including Eton and Windsor. The War Office, it seems, does not object to the hospital, but desires the gravel on which the building stands! Some military construction is proposed, for which the gravel would be useful. The Council replies that the buildings are worth twice the value of the gravel, and that gravel exists in plenty not only beneath the hospital, but all round. It certainly seems absurd, since the hospital itself is not alleged to be in the way, to suppose that all the gravel is concentrated beneath the building and that the soil is of a miraculously different nature a few yards away. The power to commandeer often produces acute intellectual laziness, and this may be true of the case in point.

### THE ALLEGED EFFECT OF STATE SUBSIDIES IN AUSTRALIA.

LAST year Mr. H. C. Malcolm, Inspector of Charities, made some scathing remarks upon the conduct of the Hamilton Hospital, Victoria, whereby the Government allotment was reduced by the Treasurer. The hospital committee appealed to the (then) Premier (Sir A. Peacock), who stultified his officer by restoring the balance. Mr. Malcolm returns to his general charges in his report for the year ending June 30, 1917, laid before State Parliament on December 18, 1917. He remarks: "The greatest defect in the management of subsidised hospitals is the practice which has been growing steadily of admitting patients who are able to pay for their treatment outside, and are in no way in need of charity." It is still thought that Mr. Malcolm refers chiefly to the Hamilton Hospital. But he remarks that payments by in-patients have increased by 259 per cent. since 1900, indicating that those who have no claim upon charity are using the public hospitals in increasing numbers and "squeezing out" the poor for whom these institutions are intended; therefore the patients' repayments are either too great or too small, as the well-to-do evidently should pay a great deal more. The immediate effect of the report has been that the new Treasurer (Mr. W. M. McPherson) has cut down the charity grant from £134,000 (1917) to £115,000 (1918), which action has spread consternation among the hospitals, but is most likely to be altered wholly or partially.

### SUGGESTED PAYING BED SCHEME.

Mr. McPherson is so impressed by the statement of the Inspector of Charities (Mr. Malcolm), that well-to-do patients are "squeezing out" poor persons from general hospitals, that he has suggested a system of "paying beds" where the well-to-do might be accommodated and receive nursing attention and food, but be left to employ and pay their own medical attendant. Formerly the Alfred Hospital, Melbourne, maintained a number of small wards of four beds, where the charge, inclusive of medical attendance and operations, was 30s. per week; but, at the instance of the Government, these were abolished some years ago. At present the question of paying wards is under discussion by the committee.

### THE COST OF A DISTRICT NURSE.

Because of diverted subscriptions, the East London Nursing Society, which provided trained nurses for the sick poor, is obliged to part with some of its nurses. It is now fifty years since the society was formed, in consequence of the experience gained during the cholera epidemic of 1866. It claims to be the oldest society in London for district nursing, and began, after the example of Florence Nightingale, by sending three trained nurses in the charge of a matron to three districts in the East End. In 1917 this number had risen to twenty-four. The cost of this work is £3,000 a year, for a district nurse cannot be provided nowadays at less than £135 annually. Since the society began the cost of a district nurse has doubled, from which we can see how the status of the work has improved.

### HOLIDAYS AND COTTAGE HOSPITALS.

In order that the matron and the nurse of Thetford Cottage Hospital may take their holiday it has been proposed that the institution should be closed during their absence. This proposal has created dissatisfaction among the working classes of Thetford, to whom Mr. G. O. Read, the secretary, has endeavoured to explain that there is no alternative, since a qualified nurse is, he says, unobtainable, and somebody must take the matron's place. We can hardly believe that a temporary nurse is unobtainable, and evidence of the steps taken in vain to find a suitable substitute should be produced. What has Mr. Read attempted in the matter?

### NURSES' FEES IN QUARANTINE.

The nursing of infectious cases offers a difficulty to the nurse who is compelled to lose her earning power during the quarantine subsequently imposed on her. The matter was recently considered by the Central Board of the South African Trained Nurses' Association, when the following resolution was passed: "That the British Medical Association be asked to frame regulations for the quarantining of nurses after attending cases of infectious disease, and that the proper authorities be then asked to enforce such regulations upon nurses and midwives." In the discussion of this motion, which was passed, it was pointed out that there is no such thing in the Transvaal as quarantine after an infectious case. The nurse merely undergoes disinfection, and is at once ready for the next case, unless quarantine is formally recommended by the doctor. The *South African Nursing Record*, from which we take this report, urges that an understanding on quarantine periods should be arrived at through the help of the British Medical Association, and suggests that nurses must be allowed half fees during the period of compulsory idleness if their unwillingness to undertake infectious cases is to be satisfactorily overcome.

### A MASSAGE SCHOOL AT CAPE TOWN.

It is hoped that a School of Massage will soon be established at the Cape, with its own examination and certificate of proficiency.

### THIS WEEK'S DRUG MARKET.

Business is unusually dull; in fact the state of the market this week is something like that in a holiday week in normal times so far as the volume of business is concerned. There the resemblance ends, however, for present prices do not bear comparison with anything ruling during the months preceding the war. The price of sugar of milk has advanced even beyond the astonishing figures of the last few months; it is now quoted at something like fifteen times the pre-war price, and the wonder is that there should be buyers willing to pay such exceedingly high prices. Honey has not yet come under official control; there has been more disposition to operate, and the market for this article is steadier. The hope that there would have been by this time a sufficiency of British-made saccharin to meet the heavy demand has been disappointed, and makers of tablets will have to supplement their British supplies with American saccharin for some little time to come. The demand for mercurials has improved; prices are firmly maintained. Available supplies of cocaine are smaller than ever, and prices are likely to advance. A rise in makers' quotations for morphine would not come as a surprise.

### TO OUR READERS.

Contributions are specially invited from any of our readers to these columns. They should deal with topical subjects and news. They must be authenticated for the information of the Editor only. The minimum payment if published is 5s. There is no hard-and-fast rule as to space, but notes of about twenty lines in length are preferred.

